# Brain connectomes in youth at risk for serious mental illness: an exploratory analysis

**DOI:** 10.1186/s12888-022-04118-4

**Published:** 2022-09-15

**Authors:** Paul D. Metzak, Mohammed K. Shakeel, Xiangyu Long, Mike Lasby, Roberto Souza, Signe Bray, Benjamin I. Goldstein, Glenda MacQueen, JianLi Wang, Sidney H. Kennedy, Jean Addington, Catherine Lebel

**Affiliations:** 1 Department of Psychiatry, Hotchkiss Brain Institute, University of Calgary, Calgary, AB Canada; 2 Department of Psychology, St.Mary’s University, Calgary, AB Canada; 3 Mathison Centre, 3280 Hospital Dr NW, Calgary, AB T2N 4Z6 Canada; 4 Department of Radiology, University of Calgary, Calgary, AB Canada; 5 Department of Radiology, Alberta Children’s Hospital Research Institute, Calgary, AB Canada; 6 Department of Radiology, Child and Adolescent Imaging Research Program, Calgary, AB Canada; 7 Department of Electrical and Software Engineering, University of Calgary, Calgary, AB Canada; 8 Centre for Youth Bipolar Disorder, Center for Addiction and Mental Health, Toronto, ON Canada; 9 Department of Psychiatry, Faculty of Medicine, University of Toronto, Toronto, ON Canada; 10 Department of Pharmacology, Faculty of Medicine, University of Toronto, Toronto, ON Canada; 11 Department of Community Health and Epidemiology, Faculty of Medicine, Dalhousie University, Nova Scotia, Canada; 12 Department of Psychiatry, University Health Network, Toronto, ON Canada; 13 Department of Psychiatry, St. Michael’s Hospital, Toronto, ON Canada; 14 Arthur Sommer Rotenberg Chair in Suicide and Depression Studies, St. Michael’s Hospital, Toronto, ON Canada; 15 Li Ka Shing Knowledge Institute, St. Michael’s Hospital, Toronto, ON Canada; 16 Krembil Research Institute, University Health Network, Toronto, ON Canada

**Keywords:** Transdiagnostic risk, Connectome, Structural connectivity, Functional connectivity, PROCAN, Major depressive disorder, Constrained spherical deconvolution, Resting-state fMRI, Linear support vector machine

## Abstract

**Background:**

Identifying early biomarkers of serious mental illness (SMI)—such as changes in brain structure and function—can aid in early diagnosis and treatment. Whole brain structural and functional connectomes were investigated in youth at risk for SMI.

**Methods:**

Participants were classified as healthy controls (HC; *n* = 33), familial risk for serious mental illness (stage 0; *n* = 31), mild symptoms (stage 1a; *n* = 37), attenuated syndromes (stage 1b; *n* = 61), or discrete disorder (transition; *n* = 9) based on clinical assessments. Imaging data was collected from two sites. Graph-theory based analysis was performed on the connectivity matrix constructed from whole-brain white matter fibers derived from constrained spherical deconvolution of the diffusion tensor imaging (DTI) scans, and from the correlations between brain regions measured with resting state functional magnetic resonance imaging (fMRI) data.

**Results:**

Linear mixed effects analysis and analysis of covariance revealed no significant differences between groups in global or nodal metrics after correction for multiple comparisons. A follow up machine learning analysis broadly supported the findings. Several non-overlapping frontal and temporal network differences were identified in the structural and functional connectomes before corrections.

**Conclusions:**

Results suggest significant brain connectome changes in youth at transdiagnostic risk may not be evident before illness onset.

**Supplementary Information:**

The online version contains supplementary material available at 10.1186/s12888-022-04118-4.

## Background

Most serious mental illnesses (SMI)—like schizophrenia, bipolar disorder, and major depressive disorder (MDD)—begin in adolescence [1] and can result in impaired quality-of-life, medical morbidity, and increased suicidality. Identifying biomarkers—such as changes in brain structure and function—may aid in early and accurate identification of individuals at risk for SMI. However, current studies on brain-based biomarkers are limited by three factors.

First, most studies have focused on individuals at clinical high risk for psychosis (CHR) rather than transdiagnostic models. For instance, studies have found that abnormalities in the modular organization of the functional connectome [2], decreased network efficiency, and disrupted small-worldness [3] are predictive of transition to psychosis in CHR. However, early prodromal phase symptoms are not well-distinguished, and CHR studies do not consider the range of phenotypic and functional outcomes that may be present in early phase of illnesses [4–6]. Therefore, there is increasing consensus that broader transdiagnostic approaches may be more suitable to investigate risk for SMI [7].

Second, studies have often focused on specific tracts or regions of interest [8]. Given that most SMI involve disrupted communication involving several brain regions [9], a far more useful approach may be to investigate whole brain connectivity networks. Aberrant connectivity has been observed in almost all major mental disorders, and disruption in these circuits often results in susceptibility to broad domains of psychopathology rather than discrete disorders, providing further support for the use of transdiagnostic over discrete disorder models [10].

Finally, no studies so far have investigated both structural as well as functional connectivity in the same sample of individuals at transdiagnostic risk, despite evidence that simultaneous analyses of functional and structural networks may provide complementary insights into brain organization for psychopathology [11].

In the current study, we used a closely matched data analysis pipeline to investigate whole brain structural and functional connectomes in a sample of individuals at transdiagnostic risk for SMI compared to controls. We followed this up with a machine learning (ML) analysis of the data to determine whether linear support-vector machine (SVM) analysis can identify combinations of features which may help distinguish between the groups. As this is the first study of its kind, we abstained from proposing specific hypotheses as there are no previous studies on which to base our predictions. Most previous neuroimaging studies have either conceptualized transdiagnostic risk differently (e.g., [12]) or focused on CHR classification. However, CHR findings cannot be used as hypotheses for transdiagnostic studies owing to the significant difference between the two approaches as well as differences in the conditions to which participants transition.

## Methods

### Participants

Participants for the current study were recruited from the larger Canadian Psychiatric Risk and Outcome study (PROCAN), which investigates youth at risk for SMI and consists of participants from the University of Calgary and Sunnybrook Health Sciences Centre [13, 14]. Participants were included in the study if they were 12–25 years of age, had an IQ > 70, did not meet criteria (at baseline) for a SMI or any medical condition affecting the central nervous system, and met one of the staging criteria [15]. To determine clinical stage assignment, a consensus based decision-making process was used. Participants who presented with familial risk factors (e.g., having a first degree relative with a psychiatric condition) but were asymptomatic were categorized as stage 0. Participants who presented with mild anxiety or depressive symptoms were classified as stage 1a, and participants who presented with attenuated syndromes were classified as stage 1b. The terms stage 0, stage 1a, and stage 1b will be used here to refer to familial risk for serious mental illness, mild symptoms, and attenuated syndromes, respectively. A group of healthy controls (HC) with no personal or family history of mental illness was also recruited for comparison. None of the healthy controls or stage 0 participants met any of the criteria for stages 1a and 1b. All participants were monitored over time to determine transition to SMI. Participants who went on to meet criteria for a SMI during a 12-months follow-up period were put into the transition group for analysis, instead of the group based on baseline symptom level (Table 1; Supplementary Table 1).

**Table 1:** Clinical staging framework for mental health disorders (Hickie et al., [15])

Stage	Definition
0	*No clinical symptoms*
	Increased risk of disorder (family history); or developmental disorder
1a	*Distress disorder*
	No attenuated psychotic symptoms
	Non-specific symptoms of anxiety or depression
	Mild to moderate severity of symptoms
	May include subjective/objective evidence of mild cognitive deficits
	Evidence of only recent or mild impacts of illness on social, educational, or occupational functioning
	May include those with earlier neurodevelopmental or attentional disorders
	who now present with anxiety or depressive symptoms
1b	*Attenuated syndromes*
	Distress disorder plus at least one moderate to severe attenuated psychotic symptom (unusual thoughts, suspiciousness, perceptual abnormalities, grandiosity, disorganization)
	Specific symptoms of anxiety or depression, brief hypomania, or brief psychotic phenomena
	May meet criteria of psychosis-risk syndromes
	May present with subthreshold manic symptoms
	May include subjective/objective evidence of at least moderate cognitive change
	Moderate to severe impact of illness on social, educational, or occupational functioning
2	*Discrete disorders*
	Discrete episodes of psychosis, mania or severe depression
	Full threshold disorder with moderate to severe symptoms and persistence over time
	Major impact of illness on social, educational, or occupational functioning
Stages not relevant for this project	
3a-3c	Incomplete remission to multiple relapses
4	Unremitting course of illness

Participant and imaging details have been described previously [13, 14, 16]. Briefly, participants were recruited from 2 sites—University of Calgary (Calgary) and Sunnybrook Health Sciences Centre (Toronto) from the larger PROCAN project. Of the 243 participants in PROCAN, 11 participants transitioned to a SMI over 12 months (all from the Calgary site): 10 participants met criteria for major depressive disorder (MDD) and 1 participant met criteria for bipolar disorder.

Imaging data was available for 173 participants, including 9 transition participants (all of whom met criteria for MDD and all of whom were female). Two participants were excluded for poor quality data, bringing the final sample for analysis in the current study to 171 participants (140 from University of Calgary and 31 from Sunnybrook Health Sciences Centre). For demographic and clinical details of all participants included in the current paper, see Table 2.

**Table 2:** Demographic and clinical characteristics at baseline (*n* = 171)

		Controls	Stage 0	Stage 1a	Stage 1b	Transition	Test
*N*		33	31	37	61	9	
				*Mean (SD)*			*F*
Age (years)		19.15 (3.78)	18.13 (4.21)	18.62 (3.62)	17.08 (3.26)	16.33 (2.34)	2.61*
Education (years)		12.48 (3.41)	11.45 (3.35)	11.43 (2.54)	10.34 (2.51)^a^	10.56 (1.81)	3.02*
				*N (%)*			*χ*^*2*^
Sex	Male	15 (45.5)	11 (35.5)	15 (40.5)	29 (47.5)	0 (0)	8.00
	Female	18 (54.5)	20 (64.5)	22 (59.5)	32 (52.5)	9 (100)	
Race	Caucasian	17 (51.5)	25 (80.6)	23 (62.2)	40 (65.6)	7 (77.8)	11.53
	Asian	6 (18.2)	3 (9.7)	6 (16.2)	6 (9.8)	0 (0)	
	Interracial	4 (12.1)	3 (9.7)	5 (13.5)	9 (14.8)	1 (11.1)	
	Other	6 (18.2)	0 (0)	3 (8.1)	6 (9.8)	1 (11.1)	
Marital status	Single	33 (100)	27 (87.1)	37 (100)	57 (93.4)	9 (100)	9.06
	Other	0 (0)	4 (12.9)	0 (0)	4 (6.6)	0 (0)	
Living with	Parents	24 (72.7)	21 (67.7)	29 (78.4)	53 (86.9)	8 (88.9)	5.96
	Other^1^	9 (27.3)	10 (32.3)	8 (21.6)	8 (13.1)	1 (11.1)	
Student	No	6 (18.2)	4 (12.9)	11 (29.7)	11 (18)	1 (11.1)	3.88
	Yes	27 (81.8)	27 (87.1)	26 (70.3)	50 (82)	8 (88.9)	
Employed	Yes	16 (48.5)	11 (35.5)	20 (54.1)	15 (24.6)	4 (44.4)	10.40*
	No	17 (51.5)	20 (64.5)	17 (45.9)	46 (75.4)	5 (55.6)	

^1^Includes living with spouse/partners, living on own, living with friends, in a boarding/group home, or academic residence

^*^p < 0.05

^a^Significantly differs from controls

### Measures

Assessment measures have been described in detail elsewhere [13, 14] (Supplementary Table 1). Briefly, measures used to determine stage of risk included: Structured Interview for Psychosis Risk Syndromes [17], Scale of Psychosis-Risk Symptoms (SOPS) [18], Kessler 10 Distress Scale [19], and Quick Inventory of Depressive Symptoms [20]. The Structured Clinical Interview for DSM-5 (SCID-5) [21] was used to confirm transition to a SMI by 12 months. Transition to MDD was defined here as the presence of more than one major depressive episode.

### MRI acquisition

Complete image acquisition details including scanner model and software version, coil details, software used, and standardized scanning protocols have been described previously [22] and are described in brief here.

Imaging data were acquired on a GE 3.0 T Discovery MR750 (University of Calgary) or Philips 3.0 T Achieva scanner (Sunnybrook Health Sciences Centre). To minimize scanner differences, reference protocols were established for each site and scanner type. All data was visually inspected by expert quality-control raters, and any scans with artifacts were removed before analysis.

#### Structural imaging

Diffusion imaging data were acquired using single shot spin echo echo-planar imaging sequence on a GE 3.0 T Discovery MR750 (University of Calgary) or Philips 3.0 T Achieva scanner (Sunnybrook Health Sciences Centre). Diffusion sensitizing gradients (b = 1000 s/mm^2^) were applied along 45 (University of Calgary) and 30 (Sunnybrook Health Sciences Centre) noncollinear directions. Eight images without diffusion weighting (*b* = 0 s/mm^2^) were acquired. Isotropic 2.2 mm voxels were acquired (resampled to 0.86 mm in-plane), FOV = 220 × 220 mm, matrix = 256 × 256, TR = 8000 ms, TE = 94 ms, flip angle = 90°, anterior–posterior phase encoding. Both sites used image space reconstruction [GE ASSET (University of Calgary) and Philips SENSE (Sunnybrook Health Sciences Centre)]. Total acquisition time was 7 min and 12 s at University of Calgary and 5 min and 15 s at Sunnybrook Health Sciences Centre.

#### Functional imaging

For the University of Calgary site, the fMRI acquisition used gradient echo EPI with the following parameters: TR/TE (ms) = 2000/30; FOV = 256; flip angle = 75, pixel bandwidth = 7812.50 Hz; matrix dimensions = 64 × 64; voxel dimensions (mm) = 4 × 4 × 4; Number of slices = 36 (bottom-up interleaved).

For the Sunnybrook Health Sciences Centre scans, the fMRI acquisition used gradient echo EPI with the following parameters: TR/TE (ms) = 2000/30; FOV = 256; flip angle = 75, pixel bandwidth = 3589 Hz; matrix dimensions = 64 × 64; voxel dimensions (mm) = 4 × 4 × 4; number of slices = 36 (bottom-up interleaved).

#### Structural connectome analysis

DTI data was visually checked using FSL [23] and ExploreDTI v4.8.6 [24]. Data was pre-processed in ExploreDTI to correct for subject motion and eddy current distortions, with diffusion vectors rotated as required and automatic background masking applied [25]. Two participants were excluded owing to poor quality data (lack of fully connected DTI data) before arriving at the current sample of 171.

Tractography analyses was run in ExploreDTI. Automated whole brain constrained spherical deconvolution (CSD) was performed using a white matter mask derived from diffusion data for each participant in native space. The minimum fractional anisotropy (FA) threshold was set to 0.20 to initiate and continue tracking, and the angle threshold was set to 30°.

In order to build the individual DTI-based structural connectivity matrices, the Automated Anatomical Labeling (AAL) template from MRIcron [26] was used to subdivide the brain into 90 regions excluding the cerebellum [27, 28]. The AAL template and whole-brain fiber tractography were used as inputs to create a 90 × 90 region-wise connectivity matrix for each individual with the “PASS” option, which means 2 regions are considered to be connected even if a third region passed through [29]. Each element of the matrix contained the averaged FA value within the connected fiber tracts between regions and was set to zero if there was no connection [30]. This weighted connectivity matrix was binarized for the calculations of the graph theoretical metrics.

#### Functional connectome analysis

All T1-weighted structural images and resting state fMRI (rs-fMRI) scans were visually examined for artifacts or distortions prior to processing. The data were processed using AFNI, FSL and REST [23, 31, 32]. The T1 images from each participant were skull stripped and co-registered to their fMRI images prior to being parcellated into grey matter, white matter, and cerebrospinal fluid (CSF). The first five volumes from each of the rs-fMRI scans were removed to ensure signal stabilization, leaving a total of 295 volumes. The resting state scans then underwent correction for slice timing and head movement. The average relative framewise displacement (FD) was calculated for each participant [33]. Furthermore, as per [34], a 36-parameter matrix was generated from each participant’s rs-fMRI data. This matrix included the averaged signals from the individual whole brain mask, CSF mask, white matter mask, the six head motion parameters, and their temporal derivatives and quadratic term signals. Then a spike matrix was created using any volumes with a high FD (> 0.3 mm) [35]. These two matrices were combined, and their effects were regressed out of the rs-fMRI data. The rs-fMRI scans were then normalized to the MNI152 2009a non-linear symmetric atlas (https://www.bic.mni.mcgill.ca/ServicesAtlases/ICBM152NLin2009), band pass filtered between 0.009 and 0.08 Hz, linear trends were removed, and finally the scans were smoothed using a 4 mm full width at half max Gaussian kernel.

Slice timing, head motion correction, T1-weighted image segmentation, head motion outlier detection, co-registration, and spatial normalization and smoothing were done in FSL 6.0.3 [23]. Regression of the nuisance signals, band-pass filtering, and linear trend removal were done using AFNI version 18.0.13 [31].

The same AAL template [27, 28] that was employed for the DTI analysis was used to parcellate the rs-fMRI images into the 90 × 90 region-wise connectivity matrices used in the graph theory analyses. Each element of the raw connectivity matrix contained the averaged correlation value of the blood oxygenation level dependent (BOLD) signal fluctuations between regions. For this analysis, all negative correlations were set to zero and positive correlations were thresholded using a *p*-value of *p* < 0.05. All positive correlations weaker than the threshold value were set to zero, and all positive correlations greater than, or equal to, the threshold were set to one. All connectivity matrices were fully connected.

### Graph theory metrics

Graph theoretical metrics for global and nodal metrics were calculated from each individual connectivity matrix using the GRETNA toolbox [36] (for detailed description of metrics see [9, 28, 36–41]).

#### Global metrics

*Assortativity* is the tendency of nodes to link those nodes with similar number of edges. *Hierarchy coefficient* identifies the presence of hierarchical organization in a network.

*Small-worldness* refers to the property of combining high levels of local clustering among nodes of a network and short paths that globally link all nodes of a network. Small-worldness analysis [39] used 100 randomly-generated networks: *Clustering coefficient* measures the extent of local clustering of a network; γ measures the ratio between the real clustering coefficient and that of the random networks; λ measures the ratio between the real shortest path length and that of the random networks; *Shortest path length* quantifies the mean distance or routing efficiency between a node and all the other nodes in the network; σ is the ratio between λ and γ.

*Synchronization* measures the likelihood that all nodes fluctuate in the same wave pattern. *Global efficiency* measures the efficiency of information propagation through the whole network. *Local efficiency* assesses the efficiency of information propagation over a node’s direct neighbors.

*Intensity* measures the mean strength of the connectivity matrix by averaging all elements in the weighted matrix. For the functional analysis, this done with averaging only the positive connections that were included in the connectome.

*Density* is the ratio between the number of existing edges in the connectome and the size of the matrix.

#### Modular interactions

*Modularity* indicates the extent to which a network is organized into modules or communities with dense connections within them but sparse connections between them.

#### Nodal metrics

*Betweenness centrality* of a node measures its effect on information flow between other nodes. *Degree centrality* measures the number of the connections directly to a node. *Nodal clustering coefficient* refers to the extent of interconnectivity among neighbors of an index node. *Nodal efficiency* indicates how efficiently an index node communicates with the other nodes. Nodal local efficiency measures how efficient the communication is among the first neighbors of a node when it is removed. *Participant coefficient* is the ability of an index node to keep the communication between its own module and other modules. In this study we use the normalized participant coefficient, which corrects for the effects of the number of modules.

The whole-brain averaged metrics are the mean of each metric across all nodes of the graph.

### Machine learning analysis

Traditional supervised ML techniques were used to analyze the combined structural and functional data. The structural and functional connectivity data was combined into a single, tabular dataset consisting of 1359 features from 171 total participants (Supplementary Fig. 1). These features consisted of the connectivity metrics noted above from both the structural and functional imaging data in addition to covariates age, head movement, and imaging site.

Traditional ML techniques were used due to their interpretability and performance on datasets with a limited number of samples. We selected a linear (SVM) classifier as our model. Multi-class linear SVM classifiers define a set of hyperplanes in feature space which maximizes the margin between the various class labels. A multi-class linear SVM model classifies a given data point by selecting the class whose decision boundary hyperplane is furthest from the data point within feature space. Linear SVM remain effective in high-dimensionality feature spaces and have been used to classify connectivity data in past studies [42–45]. A one-verses-rest multi-class strategy was employed, with one model trained per category label.

We employed a leave-one-out (LOO) cross-validation methodology to train and test our models. In LOO cross-validation, the model is trained on the entire dataset except a single hold-out test sample. Each sample is tested once and the model is evaluated by considering the accuracy of the model across all folds. The data preprocessing and hyperparameter tuning was performed within each cross-validation fold to ensure that transformation of the training data was not biased by the test sample.

The dataset labels were highly imbalanced, owing to differences in group size. To address this imbalance, we upscaled under-represented categories during training such that each category had an equal number of samples. After upscaling, we applied a Yeo-Johnson power transform [46] to remove skewness and transform the input data into more Gaussian-like distributions. A z-score standard scaler was also applied following the power transform to normalize the data to a zero mean with unit variance.

Due to the high dimensionality of the data compared to the number of samples available, we pre-fit a linear SVM classifier model to the training data to perform feature selection. We performed feature selection using a slightly modified normal-based criterion as described in [47].

Features were selected based on the L1 norm of the feature weights from each label’s model divided by the L1 norm of feature weights across all features and labels to obtain a normalized weight per feature. We selected any features with weights greater than or equal to 0.1% of the sum of feature weights for our hyperparameter tuning, training, and validation steps. Since the feature weights vector describes the normal of the hyperplane decision boundaries, normal-based feature selection selects those features which significantly influence the width of the margin between hyperplanes and their associated support vectors.

A hyperparameter grid search was performed for each fold to select the optimal regularization parameter. A regularization parameter (C) of 0.1 was selected in 130 out of 171 folds, providing high regularization strength.

The source code used for our machine learning analysis is publicly available [48].

### Statistical analysis

Data analysis procedures were similar for both structural and functional data. Data was analyzed using IBM SPSS version 26 [49] and the GRETNA toolbox [36]. A combination of linear mixed effects (LME) and analysis of covariance (ANCOVA) procedures was used to test group differences on global and nodal metrics, controlling for site and age. LME was used for global metrics and modular interactions as it provides better control for site, while ANCOVA was used for nodal metrics owing to the large number of comparisons. As there was no variability in sex in the transition group (100% female), sex was not included as a covariate.

For all global metrics and modular interactions, LME analysis were run with site as a random variable throughout the analysis. For each metric, three models were developed, with factors added at every stage. Model improvement was tested using chi-square tests of -2 log-likelihood (-2LL) values estimated using maximum likelihood (ML). The initial model with no predictors (model 0) was compared to the model with age as a fixed factor (model 1) to observe whether global metrics changed as a function of age for the entire sample. After this, group and age were added as fixed factors in model 2, and model improvement was tested again (in the functional connectivity analysis, head motion, which was quantified using the relative estimated mean displacement from FSL MCFLIRT, was also included along with age in models 1 and 2 as a fixed factor). Uncorrected values are reported, but results were only interpreted if they were significant after false discovery rate (FDR) correction for multiple comparisons.

To examine separate brain networks (or modules) [28], the 90 AAL regions were clustered based on the seven networks described in Yeo et al. [50]: the visual, somatomotor, dorsal attention, ventral attention, limbic, frontoparietal, and the default mode networks. The bilateral caudate, putamen, pallidum, and thalamus were clustered to form an eighth network named the deep gray matter network [51]. Modular interactions (i.e., total number of edges) between (28 comparisons) and within (8 comparisons) these networks, and the participation coefficient based on the network parcellation, were calculated and compared between groups with LME analysis as previously described. Uncorrected values are reported, but results were only interpreted after FDR correction for multiple comparisons.

For structural and functional nodal metrics, analysis of covariance (ANCOVA) with FDR correction was preferred over LME owing to the large number of variables (90 brain regions) per comparisons. Age and site were included as covariates; head motion was also included as a covariate for functional connectivity analysis. Uncorrected values are reported, but results were only interpreted if they were significant after FDR correction.

## Results

### Participant characteristics

Socio-demographic information for all participants is provided in Table 2. There were significant differences in age between groups. There were also differences in education, though this can be explained by the age differences (younger participants have fewer years of education).

### Structural connectivity

**Fig. 1:**
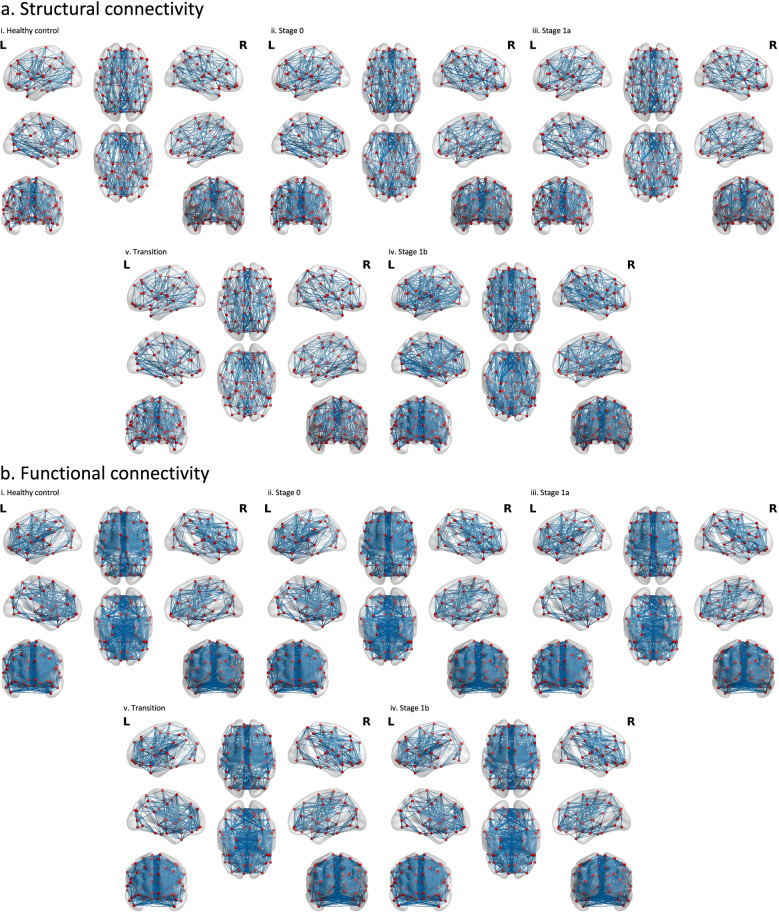
Binarized connectivity matrices from each stage of SMI risk depicted on the Montreal Neurological Institute (MNI152) brain template. Red dots represent nodes from the AAL template, and blue edges represent white matter connections between nodes for the structural analysis, and correlations that survived thresholding in the functional analysis. All figures were made using BrainNet Viewer (http://www.nitrc.org/projects/bnv/) [52]. **a** Structural connectivity. **b** Functional Connectivity

There were no significant differences between the groups on averaged connectome intensity and connectome density (Tables 3 and 4; Fig. 1a).

**Table 3:** Global metrics (mean and standard deviation) by group for structural connectivity

	Healthy controls	Stage 0	Stage 1a	Stage 1b	Transition
*n*	33	31	37	61	9
Connectome density	0.41 (0.06)	0.41 (0.05)	0.41 (0.06)	0.39 (0.04)	0.41 (0.05)
Connectome intensity	0.45 (0.03)	0.44 (0.03)	0.44 (0.03)	0.45 (0.02)	0.44 (0.01)
Assortativity (z-score)	6.26 (3.08)	6.53 (2.97)	5.94 (2.78)	4.85 (1.81)	5.93 (2.17)
Hierarchy (z-score)	5.44 (1.72)	5.05 (1.12)	5.42 (1.72)	5.44 (1.45)	5.60 (1.26)
*Network efficiency*					
Global	0.70 (0.034)	0.70 (0.026)	0.69 (0.032)	0.69 (0.026)	0.70 (0.025)
Local	0.82 (0.019)	0.82 (0.017)	0.81 (0.018)	0.81 (0.012)	0.82 (0.01)
*Small world*					
Clustering coefficient	0.64 (0.037)	0.64 (0.034)	0.64 (0.035)	0.63 (0.022)	0.63 (0.024)
ϒ (Gamma)	1.33 (0.18)	0.32 (0.12)	1.34 (0.15)	1.39 (0.14)	1.33 (0.12)
λ (Lambda)	1.00 (0.003)	1.01 (0.004)	1.01 (0.006)	1.01 (0.006)	1.00 (0.004)
Shortest path length	1.44 (0.076)	1.43 (0.053)	1.44 (0.067)	1.46 (0.056)	1.44 (0.054)
σ (Sigma)	1.32 (0.17)	1.32 (0.12)	1.32 (0.144)	1.38 (0.13)	1.33 (0.12)
Synchronization (z-score)	-11.75 (6.36)	-9.94 (7.40)	-11.15 (6.75)	-9.66 (8.44)	-14.01 (6.60)

**Table 4:** Linear mixed effects analysis for structural connectivity global metrics. Exact values reported, but findings are interpreted only if significant after false discovery rate corrections

	Model 0	AIC	BIC	Model 1	AIC	BIC	*χ*^*2*^	Sig	Model 2	AIC	BIC	*χ*^*2*^	Sig
	-2LL			-2LL			*change*		-2LL			*change*	
Connectome density	-546.75	-540.75	-531.32	-556.60	-524.60	-474.33	-9.85	n.s	-560.19	-520.19	-457.35	-3.59	n.s
Connectome intensity	-788.53	-782.53	-773.11	-811.86^a^	-779.86	-729.60	-23.33	0.05	-816.87	-776.87	-714.04	-5.01	n.s
Assortativity (z-score)	784.49	790.49	799.91	763.20	795.20	845.47	-21.29	n.s	755.34	795.34	858.17	-7.86	n.s
Hierarchy (z-score)	621.46	627.46	636.88	613.25	645.25	695.52	-8.21	n.s	610.69	650.69	713.52	-2.56	n.s
*Network efficiency*													
Global	-732.60	-726.60	-717.17	-742.24	-710.24	-659.97	-9.64	n.s	-745.92	-705.92	-643.09	-3.68	n.s
Local	-982.91	-976.91	-967.48	-1001	-969	-918.73	-18.09	n.s	-1001.85	-961.85	-899.02	-0.85	n.s
*Small world*													
Clustering coefficient	-759.99	-753.99	-744.56	-777.83	-745.83	-695.56	-17.84	n.s	-778.59	-738.59	-675.75	-0.76	n.s
ϒ (Gamma)	-164.63	-158.63	-149.20	-174.73	-142.73	-92.47	-10.10	n.s	-179.82	-139.82	-76.99	-5.09	n.s
λ (Lambda)	-1317.11	-1311.11	-1301.70	-1334.67	-1302.67	-1252.50	-17.56	n.s	-1339.10	-1299.10	-1236.38	-4.43	n.s
Shortest path length	-466.93	-460.93	-451.50	-477.080	-445.080	-394.81	-10.15	n.s	-480.99	-440.99	-378.16	-3.91	n.s
σ (Sigma)	-189.12	-183.12	-173.70	-198.99	-166.99	-116.73	-9.87	n.s	-203.93	-163.93	-101.09	-4.94	n.s
Synchronization (z-score)	1162.38	1168.38	1177.80	1148.88	1180.88	1231.15	-13.5	n.s	1143.95	1183.95	1246.79	-4.93	n.s

Model 0 = no fixed factors; Model 1 fixed factor = age; Model 2 fixed factor = age, group

^a^Significant effect of age

**Fig. 2:**
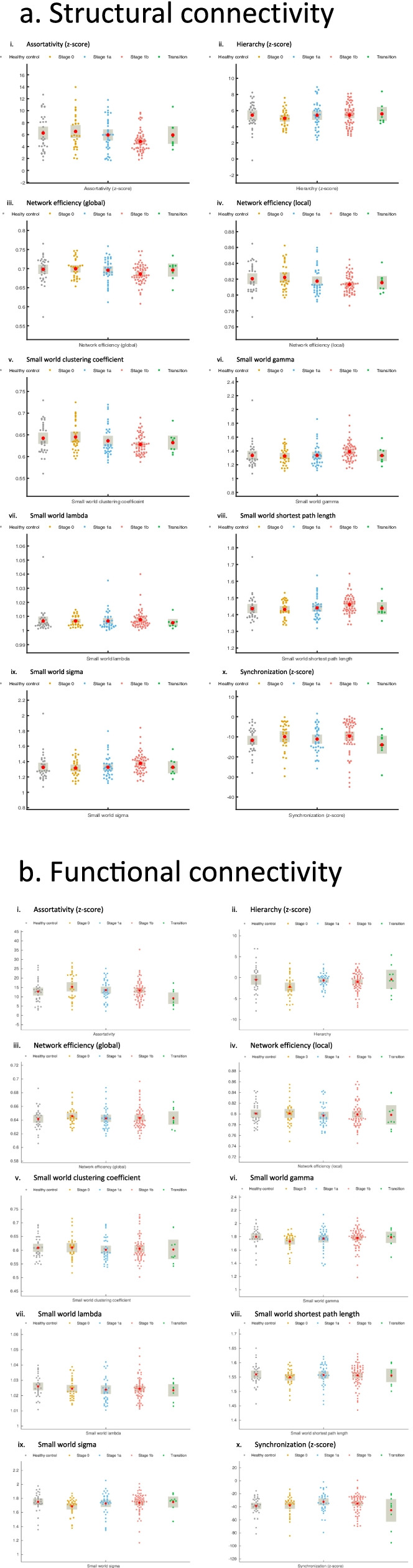
Global metrics for structural and functional connectivity. Error bars represent 95% confidence interval. **a** Structural connectivity. **b** Functional connectivity

Tables 3 and 4 show the group comparisons on whole-brain averaged metrics. There were no significant group differences on any global metrics before correction for multiple comparisons (Tables depict uncorrected values; also see Fig. 2a). Modular interactions showed a significant effect for connections between the visual and ventral attention networks, with stage 1b having lower interaction when compared to healthy controls (*p* = 0.05; Supplementary Table 2a). However, none of these effects survived FDR corrections. For nodal results (Table 5), several frontal regions (including the right dorsolateral superior frontal gyrus, left middle frontal gyrus, left superior medial orbital frontal gyrus, and right anterior cingulate) and some parietal and temporal regions (including the left angular gyrus) showed differences on nodal metrics (Supplementary Fig. 2). Participant coefficient was the most prominent metric with respect to group differences. However, none of these effects survived FDR corrections.

**Table 5:** Structural nodes with significant group differences (*p* < 0.05, uncorrected) on graph theoretical measures (also see Fig. 3a). No comparisons survived false discovery rate corrections

		Betweenness centrality		Degree centrality		Nodal cluster coefficient		Nodal efficiency		Nodal local efficiency		Nodal shortest path		Participant coefficient normalized	
Hemisphere	Anatomical Name	*F*	*p*	*F*	*p*	*F*	*p*	*F*	*P*	*F*	*p*	*F*	*p*	*F*	*p*
Right	Superior frontal gyrus, dorsolateral			2.69	0.03			2.68	0.03			2.64	0.04		
Left	Middle frontal gyrus			2.73	0.03			2.47	0.05			2.51	0.04		
Left	Olfactory bulb					2.44	0.05								
Left	Superior frontal gyrus, medial orbital					3.70	0.01			3.70	0.006	2.39	0.05	3.23	0.01
Right	Anterior cingulate and paracingulate gyri													2.56	0.04
Right	Median cingulate and paracingulate gyri													2.56	0.04
Left	Cuneus	4.26	0.003	3.29	0.01	4.40	0.002	3.35	0.01	4.45	0.002	3.36	0.01	2.70	0.03
Left	Fusiform gyrus													3.13	0.01
Left	Superior parietal gyrus					2.47	0.05			2.38	0.05				
Right	Supramarginal gyrus					2.41	0.05			2.55	0.04				
Left	Angular gyrus													3.91	0.005
Left	Lenticular nucleus putamen													3.45	0.01
Right	Heschl’s gyrus			3.18	0.01										
Right	Middle temporal pole													4.26	0.003

### Functional connectivity

No participants were removed from the analysis due to excessive motion and there were no significant differences between the stages of risk in terms of number of spikes that were censored (*F*_(4,166)_ = 1.17, *p* > 0.30).

In the functional connectome, there were no significant differences between groups in averaged connectome density or connectome intensity (Fig. 1b). Figure 2b and Tables 6 and 7 show the group comparisons on whole-brain averaged metrics from the functional imaging data. In the whole-brain averaged metrics, there were no significant group differences on any of the global metrics.

**Table 6:** Global metrics (mean and standard deviation) by group for functional connectivity

	Healthy controls	Stage 0	Stage 1a	Stage 1b	Transition
*n*	33	31	37	61	9
Connectome Density	0.32 (0.03)	0.32 (0.03)	0.32 (0.03)	0.32 (0.03)	0.32 (0.03)
Connectome Intensity	0.43 (0.03)	0.44 (0.03)	0.43 (0.03)	0.43 (0.03)	0.42 (0.04)
Assortativity (z-score)	12.86 (6.18)	15.42 (6.97)	13.64 (5.66)	13.48 (5.65)	9.25 (4.6)
Hierarchy (z-score)	-0.45 (3.39)	-2.16 (2.81)	-0.61 (1.72)	-0.97 (2.35)	-0.39 (3.38)
*Network efficiency*					
Global	0.64 (0.02)	0.65 (0.01)	0.64 (0.02)	0.64 (0.02)	0.64 (0.01)
Local	0.80 (0.02)	0.80 (0.02)	0.80 (0.02)	0.80 (0.02)	0.80 (0.03)
*Small world*					
Clustering coefficient	0.61 (0.04)	0.61 (0.04)	0.60 (0.04)	0.61 (0.05)	0.60 (0.05)
ϒ (Gamma)	1.80 (0.14)	1.73 (0.13)	1.77 (0.17)	1.78 (0.15)	1.79 (0.13)
λ (Lambda)	1.03 (0.01)	1.02 (0.01)	1.02 (0.01)	1.02 (0.01)	1.02 (0.01)
Shortest path length	1.56 (0.04)	1.55 (0.03)	1.56 (0.04)	1.56 (0.04)	1.56 (0.04)
σ (Sigma)	1.75 (0.13)	1.69 (0.12)	1.73 (0.16)	1.74 (0.14)	1.75 (0.12)
Synchronization (z-score)	-38.64 (14.21)	-37.77 (14.95)	-32.34 (15.97)	-34.98 (14.89)	-45.35 (25.61)

**Table 7:** Linear mixed effects analysis for functional connectivity global metrics. Exact values reported, but findings are interpreted only if significant after false discovery rate corrections

	Model 0	AIC	BIC	Model 1	AIC	BIC	*χ*^*2*^	Sig	Model 2	AIC	BIC	*χ*^*2*^	Sig
	-2LL			-2LL			*Change*		-2LL			*change*	
Functional connectome density	-725.28	-719.28	-709.85	-736.31^a^	-726.31	-710.60	-11.03	0.01	-737.80	-719.80	-691.52	-1.49	n.s
Functional connectome intensity	-687.19	-681.19	-671.77	-694.37^a^	-684.37	-668.66	-7.18	0.05	-697.25	-679.25	-650.97	-2.88	n.s
Assortativity (z-score)	1099.39	1105.39	1114.81	1099.28	1109.28	1124.99	-0.11	n.s	1091.17	1109.17	1137.45	-8.11	n.s
Hierarchy (z-score)	811.92	817.92	827.35	810.52	820.52	836.23	-1.40	n.s	803.03	821.03	849.31	-7.49	n.s
*Network efficiency*													
Global	-937.42	-931.42	-922.00	-951.69^a^	-941.69	-925.98	-14.27	0.001	-953.09	-935.09	-906.81	-1.40	n.s
Local	-809.46	-803.46	-794.04	-813.38	-803.38	-787.67	-3.92	n.s	-814.27	-796.27	-768.00	-0.89	n.s
*Small world*													
Clustering coefficient	-579.38	-573.38	-563.95	-582.73	-572.73	-557.02	-3.35	n.s	-583.61	-565.61	-537.33	-0.88	n.s
ϒ (Gamma)	-164.84	-158.84	-149.41	-170.04	-160.04	-144.33	-5.20	n.s	-173.39	-155.39	-127.12	-3.35	n.s
λ (Lambda)	-1184.65	-1178.65	-1169.23	-1195.13^b^	-1185.13	-1169.42	-10.48	0.01	-1198.17	-1180.17	-1151.89	-3.04	n.s
Shortest path length	-641.04	-635.04	-625.62	-654.94^a^	-644.94	-629.23	-13.90	0.001	-656.49	-638.49	-610.22	-1.55	n.s
σ (Sigma)	-188.55	-182.55	-173.12	-192.98	-182.98	-167.27	-4.43	n.s	-196.39	-178.39	-150.11	-3.41	n.s
Synchronization (z-score)	1427.38	1433.38	1442.80	1420.21^a^	1430.21	1445.91	-7.17	0.05	1411.90	1429.90	1458.18	-8.31	n.s

Model 0 = no fixed factors; Model 1 fixed factor = age, head movement; Model 2 fixed factor = age, group, head movement

^a^Significant effect of head movement

^b^Significant effect of age

There were some differences in the modular interactions from the functional analysis (all *p* = 0.05; Supplementary Table 2b). Specifically, group differences were found in the connection between the somatomotor network and the dorsal attention network, as well as the within module connections in the dorsal attention and limbic networks. None of these effects survived FDR corrections.

For nodal results, several frontal (left inferior orbitofrontal gyrus, left cingulum, and bilateral precentral gyri) and temporal regions (bilateral superior temporal poles, right parahippocampal gyrus, right superior gyrus, and right Heschl’s gyrus), as well as the right postcentral gyrus of the parietal lobe, showed differences on nodal metrics (Table 8). Participant coefficient was the most prominent metric with respect to group differences. However, none of these effects survived FDR corrections.

**Table 8:** Functional nodes with significant group differences (*p* < 0.05, uncorrected) on graph theoretical measures (also see Fig. 3b). No comparisons survived false discovery rate corrections

		Betweenness centrality		Degree centrality		Nodal cluster coefficient		Nodal efficiency		Nodal local efficiency		Nodal shortest path		Participant coefficient normalized	
Hemisphere	Anatomical Name	*F*	*p*	*F*	*p*	*F*	*p*	*F*	*P*	*F*	*p*	*F*	*p*	*F*	*p*
Left	Olfactory bulb	4.49	0.001												
Left	Inferior orbitofrontal			3.40	0.01			2.49	0.05			2.55	0.04		
Left	Cingulum			2.88	0.02										
Right	Middle temporal pole			3.16	0.02			3.02	0.02			3.32	0.01		
Right	Parahippocampal gyrus					3.72	0.01			4.02	0.004				
Left	Superior temporal pole					4.19	0.003			4.14	0.003				
Right	Superior temporal pole									2.47	0.05				
Left	Rectus											2.57	0.04		
Left	Precentral gyrus													3.50	0.01
Right	Precentral gyrus													2.74	0.03
Right	Postcentral gyus													2.63	0.04
Right	Heschl’s gyrus													2.53	0.04
Right	Superior temporal gyrus													2.82	0.03

### Machine learning analysis

**Fig. 3:**
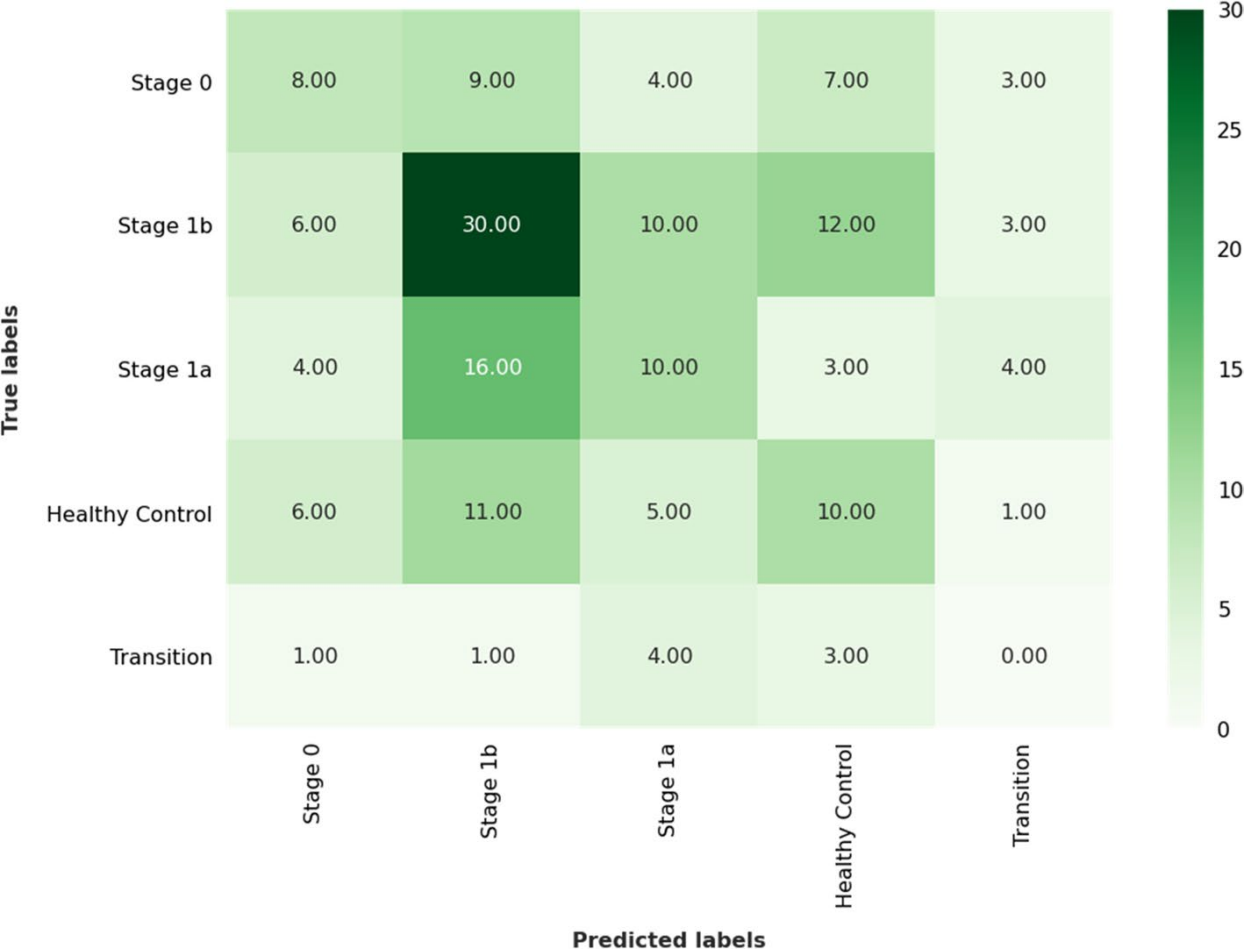
Confusion matrix of true vs. labels predicted by the linear SVM classifier using leave-one-out cross-validation

Our LOO cross-validation methodology trained and tested 171 models, one for each fold. The Linear SVM classifier had an overall accuracy and f1-score of 33.91% and 33.76%, respectively. A confusion matrix comparing true labels to predicted labels is depicted in Fig. 3. On average, 251 of 1359 features were selected for each fold. These features had weights greater than or equal to 0.1% of the L1 norm of feature weights.

**Fig. 4:**
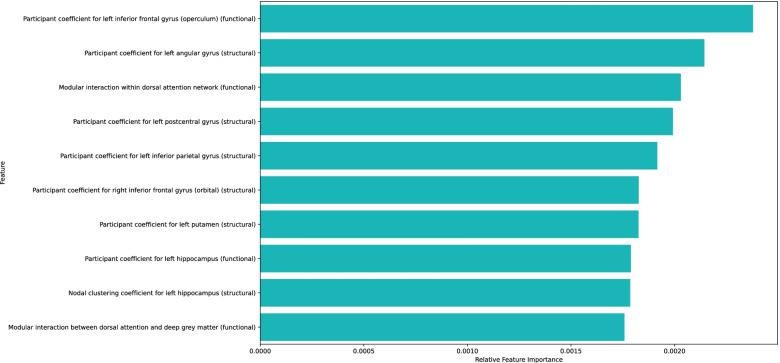
Feature versus relative feature importance based on feature weights. 7 out of 10 of the top features were comprised of participant coefficient nodal metrics

Our modified normal-based feature selection methodology was used to select features with high relative importance. The average feature importance was calculated over all classes and all cross-validation folds to determine the most important features. The top 10 most important features are depicted in Fig. 4. Similar to structural and functional findings, global metrics were not identified as significant features, while participant coefficient nodal metrics comprised 7 out of the top 10 most important features. However, only some features overlap with regions identified in structural (left angular gyrus participant coefficient) and functional findings (dorsal attention network).

## Discussion

### Structural connectivity

Here, in the first study of structural connectome in transdiagnostic risk, we found similar structural brain connectome profiles across different stages of transdiagnostic risk for SMI. However, uncorrected results may be interpreted cautiously to suggest the presence of some modular interaction differences between groups. The results showed an uncorrected significant effect for connections between the visual and ventral attention networks, with stage 1b having lower interaction than healthy controls. Similarly, nodal results may suggest several frontal regions (including the right dorsolateral superior frontal gyrus, left middle frontal gyrus, left superior medial orbital frontal gyrus, and right anterior cingulate) show between-group differences in the sample. While conclusions cannot be drawn owing to lack of significance after correction, the findings suggest specific networks and brain regions which may be the focus of future investigations.

Previous studies of MDD generally point to decreased structural connectivity, especially within frontal-subcortical networks and the default mode network [9]. One study reported reduced global efficiency and increased path length in patients with remitted geriatric depression. Depressed patients also had nodal differences from controls in several frontal regions [53]. A large study of MDD outpatients [54] found lower structural connectivity in the default mode network as well as the frontal-thalamo-caudate regions compared to controls. A third study using support vector machine based classification found that small-worldness was the most useful graph metric for classification between MDD and healthy controls [44]. They also reported degree centrality differences in the right inferior parietal cortex, and right pars orbitalis and left rostral anterior cingulate in MDD. Other studies have reported structural abnormalities in white matter regions that link prefrontal cognitive control areas with subcortical emotion processing regions [55]. On the other hand, a small number of studies of MDD have failed to find connectivity alterations [11, 56] or abnormalities in global connectivity metrics [54, 57] in MDD. Our findings suggest subtle alterations, but none survived multiple comparison correction. It is possible that transdiagnostic risk is characterized by subtle differences in frontal networks which become more prominent following pathological changes after illness onset.

### Functional connectivity

Our functional connectome results matched the structural connectome results, in that all groups showed similar profiles. Furthermore, like the structural results, the functional connectivity analysis showed group differences in modular interactions between the somatosensory network and the dorsal attention network, as well as within the limbic network and the dorsal attention network before multiple comparison correction. Pairwise comparisons between groups at differing stages of risk did not show significant differences, however, limiting our ability to draw conclusions about the cause of group differences. In the nodal results, prior to correction for multiple comparisons, group differences were found in several frontal and temporal regions, as well as the parietal region.

These findings are in-line with previous graph theoretic investigations of MDD and psychosis. A recent graph theory study using resting state fMRI data found that functional connectivity in healthy controls and unmedicated participants in their first depressive episode exhibited a similar small world regime, but differed on nodal properties in several regions including the right hippocampus [58]. Another recent study found altered nodal properties of several brain regions in MDD, including bilateral anterior cingulate, right hippocampus, and bilateral middle temporal gyri [59], which overlap with, or are adjacent to some of the regions identified in our results. Furthermore, a study examining graph theory metrics in first episode psychosis found no differences between healthy controls and those in their first episode of psychosis at baseline or at a 12 month follow-up visit [60], suggesting that the differences in graph theory properties may be difficult to detect in early phases of illness. Changes to structural metrics have been found to precede changes to functional metrics in MDD [61], so a similar process may spare functional connectome measures until later in psychosis as well.

### Machine learning

The selected linear SVM classification model was unable to effectively discriminate between the participant categories significantly beyond a random-draw baseline. The high regularization selected by the hyperparameter search suggested that the features were relatively noisy in predicting the category of each data point. However, the selection of only 18.5% of features on average as significant and the high importance rank of features derived from the participant coefficient nodal metrics suggests that these features may be of interest in future studies. Further work with a larger dataset may validate these findings.

### General discussion

The structural and functional connectome analyses used in this study both found similar structural and functional brain connectome profiles across different stages of transdiagnostic risk. The study did not find significant group differences on any of the global and nodal metrics, or modular interactions after corrections, suggesting that changes to brain structure and function may not be prominent during the at-risk phase. Uncorrected results may be interpreted cautiously to guide future research, as they suggest that subtle changes occur in frontal and attention networks in those at transdiagnostic SMI risk. Functional connectivity results additionally implicate temporal regions and suggests a possible role for the limbic network in transdiagnostic risk. While the results do not survive corrections and require validation from future studies, the differences between structural and functional findings also provide support for the view that while structural and functional networks may share similar topological mechanisms [9, 62, 63] functional connectivity changes may not be entirely constrained by differences in underlying structural connectivity [63, 64] making combined connectome approaches a valuable tool in identifying neurophysiological changes in individuals who go on to develop SMI.

### Limitations

The primary limitation of this study is the lack of power owing to the small number of individuals who transitioned to a SMI. Declining transition rates are a common problem in studies of at-risk individuals. Though numbers are lacking from transdiagnostic studies, evidence from CHR suggests that current transition rates are about 15% [65]. Despite this limitation, transdiagnostic studies remain critical to identify risk biomarkers in vulnerable populations. Future studies may benefit from multi-site collaborations or sample enrichment from studies using similar acquisition protocols (see [66]).

A second limitation of the study is the possibility of scanner differences between sites. While we used standardized acquisition protocols and statistically controlled for site using multilevel modeling, we acknowledge that scanner-induced discrepancies may still confound results.

In this study, we chose to binarize the structural and functional connectome networks, instead of using weighted networks, even though weighted networks preserve biological information better than binarization [67]. Binarized networks are relatively unaffected by connectivity strength, which allows us to directly compare information derived from structural and functional metrics. Furthermore, binarization allows us to mitigate the effect of site differences on the data, and also makes our findings directly comparable to previous studies which have used binarized networks. Future studies incorporating weighted networks into graph-theoretical analysis may help further elucidate relationships between brain networks and risks for mental illness.

Finally, follow-up for this study was limited to one year. It is likely that some of these individuals will transition to a SMI as time goes on. Future studies with longer follow-up may be better able to elucidate connectome changes associated with longer term transitions.

With respect to SVM, the relatively small sample size, unbalanced dataset, and high dimensional feature space adversely affected the linear SVM model performance. Increasing the sample size, particularly for the transition category, may yield improved results using similar machine learning methodologies.

## Supplementary Information

**Additional file 1:** **Supplementary figure 1.** Machine analysis flowchart. **Supplementary figure 2.** Structural and functional nodes with significant group differences (*p* <.05, uncorrected). Blue nodes are from the structural analysis, red nodes are from the functional analysis, and the purple nodes were found in both analyses. None of the differences survive FDR correction for multiple comparisons. See Table 2 for details.

**Additional file 2:** **Supplementary Table 1.** Detailed clinical criteria for PROCAN [13]. Participants can meet Stages 0 to 1b for entry into the study. **Supplementary Table 2a**. Linear mixed effects analysis for structural connectivity modular interactions (based on [50]). **Supplementary Table 2b.** Linear mixed effects analysis for functional connectivity modular interactions (based on [50]).

## Data Availability

The datasets generated and/or analyzed during the current study are not publicly available due to privacy and ethical restrictions but are available from the corresponding author on reasonable request.

## References

[1] Paus T, Keshavan M, Giedd JN (2008). Why do many psychiatric disorders emerge during adolescence?. Nat Rev Neurosci.

[2] Collin G, Seidman LJ, Keshavan MS, Stone WS, Qi Z, Zhang T (2020). Functional connectome organization predicts conversion to psychosis in clinical high-risk youth from the SHARP program. Mol Psychiatry.

[3] Worthington MA, Cao H, Cannon TD (2020). Discovery and validation of prediction algorithms for psychosis in youths at clinical high risk. Biol Psychiatry Cogn Neurosci Neuroimaging.

[4] Hartmann JA, Nelson B, Ratheesh A, Treen D, McGorry PD (2018). At-risk studies and clinical antecedents of psychosis, bipolar disorder and depression: a scoping review in the context of clinical staging. Psychol Med.

[5] McGorry P, Nelson B (2016). Why we need a transdiagnostic staging approach to emerging psychopathology, early diagnosis, and treatment. JAMA Psychiat.

[6] Scott J, Leboyer M, Hickie I, Berk M, Kapczinski F, Frank E (2013). Clinical staging in psychiatry: a cross-cutting model of diagnosis with heuristic and practical value. Br J Psychiatry.

[7] McGorry PD, Hickie IB (2019). Clinical Staging in Psychiatry: Making Diagnosis Work for Research and Treatment.

[8] Vijayakumar N, Bartholomeusz C, Whitford T, Hermens DF, Nelson B, Rice S (2016). White matter integrity in individuals at ultra-high risk for psychosis: a systematic review and discussion of the role of polyunsaturated fatty acids. BMC Psychiatry.

[9] Gong Q, He Y (2015). Depression, neuroimaging and connectomics: a selective overview. Biol Psychiat.

[10] Buckholtz JW, Meyer-Lindenberg A (2012). Psychopathology and the human connectome: toward a transdiagnostic model of risk for mental illness. Neuron.

[11] Jiang X, Shen Y, Yao J, Zhang L, Xu L, Feng R (2019). Connectome analysis of functional and structural hemispheric brain networks in major depressive disorder. Transl Psychiatry.

[12] Elliott ML, Romer A, Knodt AR, Hariri AR (2018). A connectome-wide functional signature of transdiagnostic risk for mental illness. Biol Psychiatry.

[13] Addington J, Liu L, Goldstein BI, Wang J, Kennedy SH, Bray S (2019). Clinical staging for youth at-risk for serious mental illness. Early Interv Psychiatry.

[14] Addington J, Liu L, Farris MS, Goldstein BI, Wang JL, Kennedy SH (2021). Clinical staging for youth at-risk for serious mental illness: a longitudinal perspective. Early Interv Psychiatry.

[15] Hickie IB, Scott EM, Hermens DF, Naismith SL, Guastella AJ, Kaur M (2013). Applying clinical staging to young people who present for mental health care. Early Interv Psychiatry.

[16] Shakeel MK, MacQueen G, Addington J, Metzak PD, Georgopoulos G, Bray S (2020). White matter connectivity in youth at risk for serious mental illness: a longitudinal analysis. Psychiatry Res - Neuroimaging.

[17] Miller TJ, McGlashan TH, Rosen JL, Cadenhead K, Ventura J, McFarlane W (2003). Prodromal assessment with the structured interview for prodromal syndromes and the scale of prodromal symptoms: predictive validity, interrater reliability, and training to reliability. Schizophr Bull.

[18] McGlashan T, Walsh B, Woods S (2010). The psychosis-risk syndrome: handbook for diagnosis and follow-up.

[19] Kessler RC, Andrews G, Colpe LJ, Hiripi E, Mroczek DK, Normand SLT (2002). Short screening scales to monitor population prevalences and trends in non-specific psychological distress. Psychol Med.

[20] Trivedi MH, Rush AJ, Ibrahim HM, Carmody TJ, Biggs MM, Suppes T (2004). The Inventory of Depressive Symptomatology, clinician rating (IDS-C) and self-report (IDS-SR), and the Quick Inventory Depressive Symptomatology, clinician rating (QIDS-C) and self-report (QIDS-SR) in public sector patients with mood disorders: A psychome. Psychol Med.

[21] First MB, Williams JBW, Karg RS, Spitzer RL (2015). Structured Clinical Interview for DSM-5—Research Version (SCID-5 for DSM-5, Research Version; SCID-5-RV).

[22] MacQueen GM, Hassel S, Arnott SR, Addington J, Bowie CR, Bray SL (2019). The Canadian Biomarker Integration Network in Depression (CAN-BIND): magnetic resonance imaging protocols. J Psychiatry Neurosci.

[23] Jenkinson M, Beckmann CF, Behrens TEJ, Woolrich MW, Smith SM (2012). FSL Neuroimage.

[24] Leemans A, Jeurissen B, Sijbers J, Jones DK. 2009. ExploreDTI: a graphical toolbox for processing, analyzing, and visualizing diffusion MR data. In: Proceedings of the International Society for Magnetic Resonance in Medicine. 3537.

[25] Leemans A, Jones DK (2009). The B-matrix must be rotated when correcting for subject motion in DTI data. Magn Reson Med.

[26] Rorden C, Brett M (2000). Stereotaxic display of brain lesions. Behav Neurol.

[27] Tzourio-Mazoyer N, Landeau B, Papathanassiou D, Crivello F, Etard O, Delcroix N (2002). Automated anatomical labeling of activations in SPM using a macroscopic anatomical parcellation of the MNI MRI single-subject brain. Neuroimage.

[28] Bullmore E, Sporns O (2009). Complex brain networks: Graph theoretical analysis of structural and functional systems. Nat Rev Neurosci.

[29] Sidlauskaite J, Caeyenberghs K, Sonuga-Barke E, Roeyers H, Wiersema JR (2015). Whole-brain structural topology in adult attention-deficit/hyperactivity disorder: preserved global - Disturbed local network organization. NeuroImage Clin.

[30] Long X, Little G, Treit S, Beaulieu C, Gong G, Lebel C (2020). Altered brain white matter connectome in children and adolescents with prenatal alcohol exposure. Brain Struct Funct.

[31] Cox RW (1996). AFNI: Software for analysis and visualization of functional magnetic resonance neuroimages. Comput Biomed Res.

[32] Song XW, Dong ZY, Long XY, Li SF, Zuo XN, Zhu CZ (2011). REST: A Toolkit for resting-state functional magnetic resonance imaging data processing. PLoS One.

[33] Jenkinson M, Bannister P, Brady M, Smith S (2002). Improved optimization for the robust and accurate linear registration and motion correction of brain images. Neuroimage.

[34] Satterthwaite TD, Elliott MA, Gerraty RT, Ruparel K, Loughead J, Calkins ME (2013). An improved framework for confound regression and filtering for control of motion artifact in the preprocessing of resting-state functional connectivity data. Neuroimage.

[35] Power JD, Schlaggar BL, Petersen SE (2014). Recent progress and outstanding issues in motion correction in resting state fMRI. Neuroimage.

[36] Wang J, Wang X, Xia M, Liao X, Evans A, He Y (2015). GRETNA: a graph theoretical network analysis toolbox for imaging connectomics. Front Hum Neurosci.

[37] Farahani FV, Karwowski W, Lighthall NR (2019). Application of graph theory for identifying connectivity patterns in human brain networks: a systematic review. Front Neurosci.

[38] Fornito A, Zalesky A, Breakspear M (2015). The connectomics of brain disorders. Nat Rev Neurosci.

[39] Maslov S, Sneppen K (2002). Specificity and stability in topology of protein networks. Science (80- ).

[40] van den Heuvel MP, Sporns O (2011). Rich-club organization of the human connectome. J Neurosci.

[41] Wang JH, Zuo XN, Gohel S, Milham MP, Biswal BB, He Y (2011). Graph theoretical analysis of functional brain networks: Test-retest evaluation on short- and long-term resting-state functional MRI data. PLoS One.

[42] Deshpande G, Libero LE, Sreenivasan KR, Deshpande HD, Kana RK (2013). Identification of neural connectivity signatures of autism using machine learning. Front Hum Neurosci.

[43] Watanabe T, Kessler D, Scott C, Angstadt M, Sripada C (2014). Disease prediction based on functional connectomes using a scalable and spatially-informed support vector machine. Neuroimage.

[44] Sacchet MD, Prasad G, Foland-Ross LC, Thompson PM, Gotlib IH (2015). Support vector machine classification of major depressive disorder using diffusion-weighted neuroimaging and graph theory. Front Psychiatry.

[45] Crimi A, Dodero L, Murino V, Sona D. 2017. Case-control discrimination through effective brain connectivity. In: Proceedings - International Symposium on Biomedical Imaging. IEEE Computer Society. 970–3.

[46] Yeo I-KNK, Johnson RA (2000). A new family of power transformations to improve normality or symmetry. Biometrika..

[47] Brank J, Grobelnik M, Milić-Frayling N, Mladenić D (2002). Feature selection using support vector machines. Manag Inf Syst.

[48] Lasby M, Souza R. Brain Connectomes in Youth at Risk for Serious Mental Illness: An Exploratory Analysis. https://github.com/mklasby/brain-connectomes-in-youth-at-risk. Accessed 2 Jun 2022. 10.1186/s12888-022-04118-4 PMC9476574 36109720

[49] IBM (2019). IBM SPSS Statistics for Windows, Version 260.

[50] Yeo BTT, Krienen FM, Sepulcre J, Sabuncu MR, Lashkari D, Hollinshead M (2011). The organization of the human cerebral cortex estimated by intrinsic functional connectivity. J Neurophysiol.

[51] Baum GL, Ciric R, Roalf DR, Betzel RF, Moore TM, Shinohara RT (2017). Modular segregation of structural brain networks supports the development of executive function in youth. Curr Biol.

[52] Xia M, Wang J, He Y (2013). BrainNet viewer: a network visualization tool for human brain connectomics. PLoS One.

[53] Bai F, Shu N, Yuan Y, Shi Y, Yu H, Wu D (2012). Topologically convergent and divergent structural connectivity patterns between patients with remitted geriatric depression and amnestic mild cognitive impairment. J Neurosci.

[54] Korgaonkar MS, Fornito A, Williams LM, Grieve SM (2014). Abnormal structural networks characterize major depressive disorder: a connectome analysis. Biol Psychiatry.

[55] Liao Y, Huang X, Wu Q, Yang C, Kuang W, Du M (2013). Is depression a disconnection syndrome? Meta- analysis of diffusion tensor imaging studies in patients with MDD. J Psychiatry Neurosci.

[56] Ajilore O, Lamar M, Kumar A (2014). Association of brain network efficiency with aging, depression, and cognition. Am J Geriatr Psychiatry.

[57] Qin J, Wei M, Liu H, Yan R, Luo G, Yao Z (2014). Abnormal brain anatomical topological organization of the cognitive-emotional and the frontoparietal circuitry in major depressive disorder. Magn Reson Med.

[58] Guo H, Cheng C, Cao XH, Xiang J, Chen JJ, Zhang KR (2014). Resting-state functional connectivity abnormalities in first-onset unmedicated depression. Neural Regen Res.

[59] Ye M, Yang T, Qing P, Lei X, Qiu J, Liu G (2015). Changes of functional brain networks in major depressive disorder: a graph theoretical analysis of resting-state fMRI. PLoS One.

[60] Ganella EP, Seguin C, Pantelis C, Whittle S, Baune BT, Olver J (2018). Resting-state functional brain networks in first-episode psychosis: a 12-month follow-up study. Aust N Z J Psychiatry.

[61] Yao Z, Zou Y, Zheng W, Zhang Z, Li Y, Yu Y (2019). Structural alterations of the brain preceded functional alterations in major depressive disorder patients: Evidence from multimodal connectivity. J Affect Disord.

[62] Hagmann P, Cammoun L, Gigandet X, Meuli R, Honey CJ, Van Wedeen J (2008). Mapping the structural core of human cerebral cortex. PLoS Biol.

[63] Honey CJ, Sporns O, Cammoun L, Gigandet X, Thiran JP, Meuli R (2009). Predicting human resting-state functional connectivity from structural connectivity. Proc Natl Acad Sci U S A.

[64] Reid AT, Lewis J, Bezgin G, Khundrakpam B, Eickhoff SB, McIntosh AR (2016). A cross-modal, cross-species comparison of connectivity measures in the primate brain. Neuroimage.

[65] Hartmann JA, Yuen HP, McGorry PD, Yung AR, Lin A, Wood SJ (2016). Declining transition rates to psychotic disorder in “ultra-high risk” clients: Investigation of a dilution effect. Schizophr Res.

[66] Shakeel MK, Hassel S, Davis AD, Metzak PD, MacQueen GM, Arnott SR (2021). White matter microstructure in youth at risk for serious mental illness: a comparative analysis. Psychiatry Res - Neuroimaging.

[67] Yeh CH, Jones DK, Liang X, Descoteaux M, Connelly A (2021). Mapping structural connectivity using diffusion MRI: challenges and opportunities. J Magn Reson Imaging.

[68] World Medical Association (2013). World Medical Association declaration of Helsinki: Ethical principles for medical research involving human subjects. JAMA - J Am Med Assoc.

