# Supplementary material for: Brain connectomes in youth at risk for serious mental illness: an exploratory analysis
Source: BMC Psychiatry. 2022 Sep 15;22:611. doi: 10.1186/s12888-022-04118-4 (PMC9476574; doi:10.1186/s12888-022-04118-4)

Supplementary figure 1. Machine analysis flowchart.


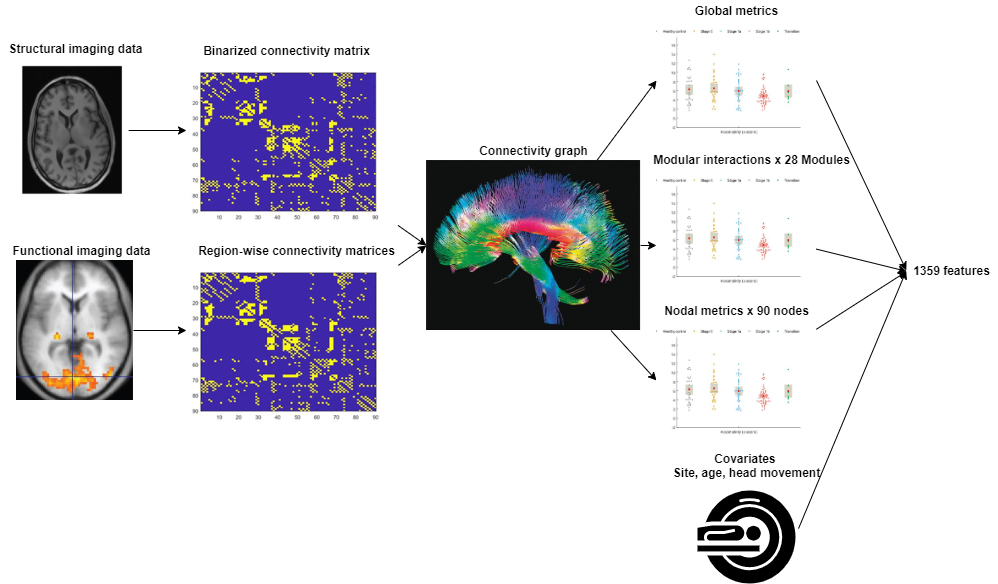


Supplementary figure 2. Structural and functional nodes with significant group differences (p <.05, uncorrected). Blue nodes are from the structural analysis, red nodes are from the functional analysis, and the purple nodes were found in both analyses. None of the differences survive FDR correction for multiple comparisons. See Table 2 for details.


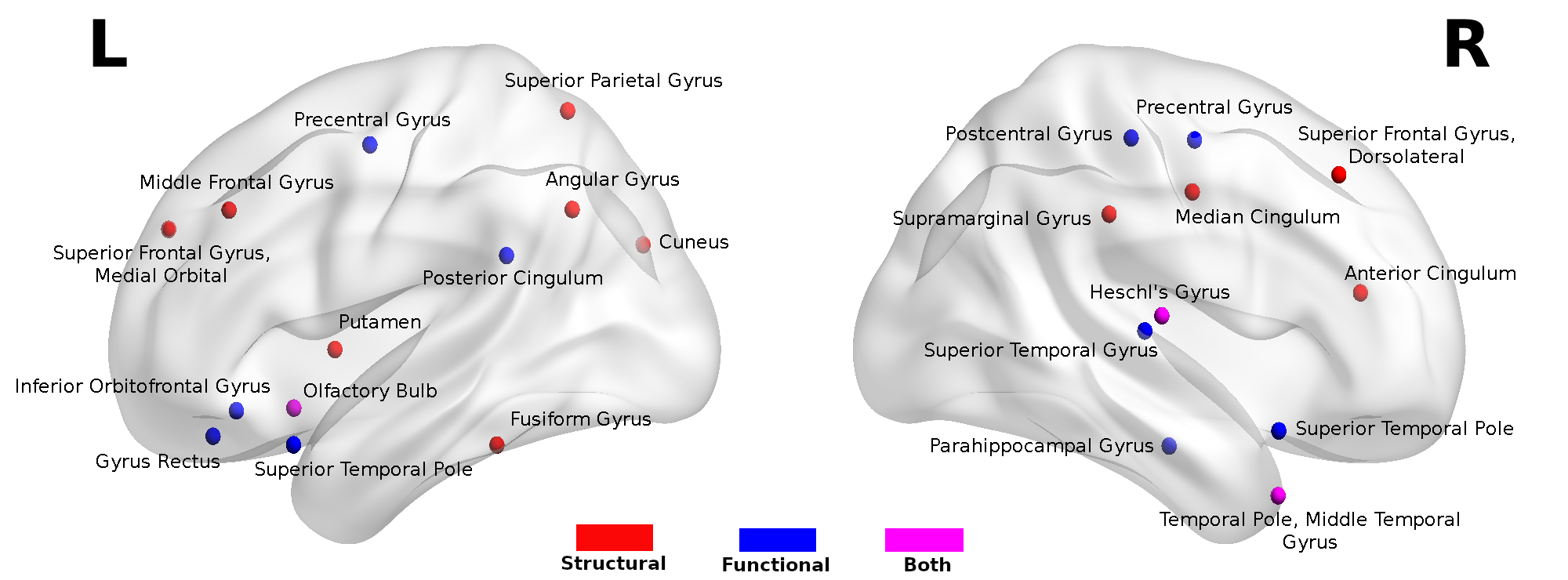

Supplement: Supplementary file 1 — Additional file 1: Supplementary figure 1. Machine analysis flowchart. Supplementary figure 2. Structural and functional nodes with significant group differences (p <.05, uncorrected). Blue nodes are from the structural analysis, red nodes are from the functional analysis, and the purple nodes were found in both analyses. None of the differences survive FDR correction for multiple comparisons. See Table 2 for details. [file 12888_2022_4118_MOESM1_ESM.docx]
