# Supplementary material for: Brain connectomes in youth at risk for serious mental illness: an exploratory analysis
Source: BMC Psychiatry. 2022 Sep 15;22:611. doi: 10.1186/s12888-022-04118-4 (PMC9476574; doi:10.1186/s12888-022-04118-4)
Supplement: Supplementary file 2 — Additional file 2: Supplementary Table 1. Detailed clinical criteria for PROCAN [13]. Participants can meet Stages 0 to 1b for entry into the study. Supplementary Table 2a. Linear mixed effects analysis for structural connectivity modular interactions (based on [50]). Supplementary Table 2b. Linear mixed effects analysis for functional connectivity modular interactions (based on [50]). [file 12888_2022_4118_MOESM2_ESM.docx]

Supplementary Table 1. Detailed clinical criteria for PROCAN (Addington, 2019). Participants can meet Stages 0 to 1b for entry into the study.

| **Stage** | **Definition** | **Clinical Features** |
| --- | --- | --- |
| 0 | **ASYMPTOMATIC INDIVIDUALS**  Subjects at increased risk of psychotic or severe mood disorder. No anxiety, depressive or psychotic  symptoms currently.  Stage 0 is not assigned to cases with symptoms presenting for assessment in healthcare settings. | Not help seeking – typically recruited from the population based on the presence of recognized risk factor to psychiatric illness.  Typically, at-risk populations specifically recruited for research based on one or more of the following:   - 1. First-degree relatives of probands   2. Family history of mental illness in multiple relatives other than first-degree (this includes participants’ reports of mental illness in family members)   3. Preterm delivery or low birthweight   4. Childhood physical or sexual abuse   5. Presence of a major developmental disorder |
| 1a | **HELP-SEEKING INDIVIDUALS WITH SYMPTOMS**  Non-specific symptoms of anxiety or depression  Symptoms may include subjective or objective evidence of mild neuropsychological deficits.  Evidence of only recent or mild impacts of illness on social, educational or occupational function. | Typically help-seeking individuals with non-specific anxiety or depressive symptoms.  For anxiety – mild to moderate levels of arousal without significant or persistent avoidant behaviours.  For depression – mild to moderate levels of depressive ideation without specific features indicative of a more disabling disorder, (e.g., 6-11 on QUIDS but no SCID diagnosis of depression)  May include those with earlier childhood-onset symptoms who have re-presented or worsened during the adolescent period.  May include those with earlier onset neurodevelopmental or attentional disorders who now present with anxiety or depressive symptoms in the adolescent years.  Typically, adolescent or early adult populations assessed in primary care or educational settings or identified by screening within relevant primary care, employment or educational settings of relevant populations. |
| 1b | **ATTENUATED SYNDROMES**  Specific symptoms of brief psychotic phenomena, brief hypomania, moderate depression, severe anxiety or presence of self-harm.  May include subjective or objective evidence of at least moderate neuropsychological change or moderate to severe impact of illness on social, education or employment functioning. | Development of more specific anxiety, depressive or mixed symptoms of at least moderate severity.  Symptoms at this stage should be persisting and clearly having a significant impact on major aspects of psychosocial function.  Comorbidity of anxiety, depressive, attenuated psychotic symptoms and substance misuse are common at this stage.  Treatment may have already commenced and/or the person may have been referred for further specialized assessment.  Some degree of treatment with an antidepressant, antipsychotic or mood-stabilizing agent is common for Individuals in this stage, particularly where there has been limited access to specialized psychological therapies.  **Clinical Stage 1b criteria:**   - 1. **Criteria of Prodromal Syndromes (COPS) criteria including longstanding symptoms**   NB. Symptoms can have been present in last year vs. begun or worsened in the past year.   - 1. **Subthreshold manic symptoms**   (A) period of abnormally and persistently elevated, expansive or irritable mood as well as at least 2 of the following (B) criteria (3 in the case of irritable mood) present at least 4 hours per day in each of at least 2 consecutive days in the last 6 months:   - Inflated self-esteem or grandiosity - Decreased need for sleep (e.g. feels rested after only three hours sleep) - Much more talkative than usual or pressure to keep talking - Flight of ideas or subjective experience that thoughts are racing - Distractibility - Increased goal directed activity (either socially, at work, or sexually) or psychomotor agitation - Excessive involvement in pleasurable activities with a high risk for painful consequences.   Duration can be 3 or less days if there is (C) unequivocal change in functioning that is uncharacteristic of the person, and (D) change in functioning is observed by others.  Duration can be up to 6 days if only C or D is met  **Exclusion.**  One week or longer of full threshold manic symptoms.   - 1. **Moderate MDD**   Current mild depressive episode (MDE) according to DSM-V  Current MDE of moderate severity (i.e. 11-15 on the QIDS)  **NB.** The following are exclusion criteria:  Current severe and persistent depression present for ≥ 6 weeks, more days than not as diagnosed on the SCID  **OR**  Up to 2 past episodes of greater severity than the current episode as determined on the SCID and/or QIDS   - 1. **Anxiety syndromes**   Characterized by more severe symptoms and development of specific avoidant behaviours   - 1. **Self-harm**   The presence of regular, deliberate self-harm without overt suicidal intent may occur in this stage. This includes impulsive low lethality overdose occurring in context of psychosocial stressor and in the absence of severe depression. |

Supplementary Table 2a. Linear mixed effects analysis for structural connectivity modular interactions (based on Yeo et al. 2011).

|  |  | Model 0 | AIC | BIC | Model 1 | AIC | BIC | *χ^2^* | Sig. (uncorrected) | Model 2 | AIC | BIC | *χ^2^* | Sig. (uncorrected) |
| --- | --- | --- | --- | --- | --- | --- | --- | --- | --- | --- | --- | --- | --- | --- |
|  |  | -2LL |  |  | -2LL |  |  | *change* |  | -2LL |  |  | *change* |  |
| *Between modules* | |  |  |  |  |  |  |  |  |  |  |  |  |  |
| 1 (visual) | 2 (somatomotor) | 1177.21 | 1183.21 | 1192.63 | 1158.60 | 1190.60 | 1240.86 | -18.61 | n.s | 1155.50 | 1195.50 | 1258.34 | -3.10 | n.s |
| 1 (visual) | 3 (dorsal attention) | 1185.12 | 1191.12 | 1200.55 | 1166.97 | 1198.97 | 1249.24 | -18.15 | n.s | 1160.64 | 1200.64 | 1263.47 | -6.33 | n.s |
| 1 (visual) | 4 (ventral attention)^a^ | 1067.59 | 1073.59 | 1083.01 | 1047.27 | 1079.27 | 1129.53 | -20.32 | n.s | 1035.02 | 1075.02 | 1137.85 | -12.25 | 0.05 |
| 1 (visual) | 5 (limbic) | 1566.01 | 1572.01 | 1581.43 | 1557.35 | 1589.35 | 1639.61 | -8.66 | n.s | 1551.94 | 1591.94 | 1654.77 | -5.41 | n.s |
| 1 (visual) | 6 (fronto-parietal) | 1336.55 | 1342.55 | 1351.97 | 1323.70 | 1355.70 | 1405.96 | -12.85 | n.s | 1316.51 | 1356.51 | 1419.35 | -7.19 | n.s |
| 1 (visual) | 7 (default mode) | 1437.41 | 1443.41 | 1452.84 | 1425.98 | 1457.98 | 1508.25 | -11.43 | n.s | 1419.71 | 1459.71 | 1522.55 | -6.27 | n.s |
| 1 (visual) | 8 (deep grey matter) | 1388.61 | 1394.61 | 1404.03 | 1380.11 | 1412.11 | 1462.38 | -8.50 | n.s | 1375.76 | 1415.76 | 1478.60 | -4.35 | n.s |
| 2 (somatomotor) | 3 (dorsal attention) | 1003.82 | 1009.82 | 1019.25 | 989.70 | 1021.70 | 1071.96 | -14.12 | n.s | 988.84 | 1028.84 | 1091.68 | -0.86 | n.s |
| 2 (somatomotor) | 4 (ventral attention) | 975.20 | 981.20 | 990.62 | 962.84 | 994.84 | 1045.10 | -12.36 | n.s | 961.21 | 1001.21 | 1064.04 | -1.63 | n.s |
| 2 (somatomotor) | 5 (limbic) | 1236.20 | 1242.20 | 1251.63 | 1225.78 | 1257.78 | 1308.05 | -10.42 | n.s | 1223.41 | 1263.41 | 1326.24 | -2.37 | n.s |
| 2 (somatomotor) | 6 (fronto-parietal) | 1116.39 | 1122.39 | 1131.81 | 1111.44 | 1143.44 | 1193.71 | -4.95 | n.s | 1105.92 | 1145.92 | 1208.76 | -5.52 | n.s |
| 2 (somatomotor) | 7 (default mode) | 1268.90 | 1274.90 | 1284.32 | 1259.88 | 1291.88 | 1342.15 | -9.02 | n.s | 1257.98 | 1297.98 | 1360.81 | -1.90 | n.s |
| 2 (somatomotor) | 8 (deep grey matter) | 1161.27 | 1167.27 | 1176.69 | 1155.53 | 1187.53 | 1237.79 | -5.74 | n.s | 1154.29 | 1194.29 | 1257.12 | -1.24 | n.s |
| 3 (dorsal attention) | 4 (ventral attention) | 682.94 | 688.94 | 689.36 | 675.58 | 707.58 | 757.85 | -7.36 | n.s | 672.30 | 712.30 | 775.14 | -3.28 | n.s |
| 3 (dorsal attention) | 5 (limbic) | 1181.52 | 1187.52 | 1196.94 | 1164.21 | 1196.21 | 1246.48 | -17.31 | n.s | 1160.39 | 1200.39 | 1263.22 | -3.82 | n.s |
| 3 (dorsal attention) | 6 (fronto-parietal) | 948.04 | 954.04 | 963.46 | 938.00 | 970.00 | 1020.27 | -10.04 | n.s | 936.36 | 976.36 | 1039.20 | -1.64 | n.s |
| 3 (dorsal attention) | 7 (default mode) | 1154.78 | 1160.78 | 1170.21 | 1136.55 | 1168.55 | 1218.82 | -18.23 | n.s | 1132.75 | 1172.75 | 1235.59 | -3.80 | n.s |
| 3 (dorsal attention) | 8 (deep grey matter) | 1049.58 | 1055.58 | 1065.00 | 1037.72 | 1069.72 | 1119.99 | -11.86 | n.s | 1033.60 | 1073.60 | 1136.43 | -4.12 | n.s |
| 4 (ventral attention) | 5 (limbic) | 1096.32 | 1102.32 | 1111.74 | 1079.47 | 1111.47 | 1161.74 | -16.85 | n.s | 1079.21 | 1119.21 | 1182.05 | -0.26 | n.s |
| 4 (ventral attention) | 6 (fronto-parietal) | 926.87 | 932.87 | 942.29 | 918.59 | 950.59 | 1000.85 | -8.28 | n.s | 914.21 | 954.21 | 1017.05 | -4.38 | n.s |
| 4 (ventral attention) | 7 (default mode) | 1075.00 | 1081.00 | 1090.43 | 1059.10 | 1091.10 | 1141.37 | -15.90 | n.s | 1058.40 | 1098.40 | 1161.23 | -0.70 | n.s |
| 4 (ventral attention) | 8 (deep grey matter) | 1035.53 | 1041.53 | 1050.95 | 1021.12 | 1053.12 | 1103.38 | -14.41 | n.s | 1018.80 | 1058.80 | 1121.63 | -2.32 | n.s |
| 5 (limbic) | 6 (fronto-parietal) | 1141.19 | 1147.19 | 1156.61 | 1132.64 | 1164.64 | 1214.90 | -8.55 | n.s | 1129.41 | 1169.41 | 1232.24 | -3.23 | n.s |
| 5 (limbic) | 7 (default mode) | 1541.19 | 1547.19 | 1556.62 | 1531.17 | 1563.17 | 1613.44 | -10.02 | n.s | 1527.59 | 1567.59 | 1630.43 | -3.58 | n.s |
| 5 (limbic) | 8 (deep grey matter) | 1331.64 | 1337.64 | 1347.07 | 1328.11 | 1360.11 | 1410.37 | -3.53 | n.s | 1324.33 | 1364.33 | 1427.16 | -3.78 | n.s |
| 6 (fronto-parietal) | 7 (default mode) | 1217.34 | 1223.34 | 1232.77 | 1196.97 | 1228.97 | 1279.24 | -20.37 | n.s | 1193.46 | 1233.46 | 1296.29 | -3.51 | n.s |
| 6 (fronto-parietal) | 8 (deep grey matter) | 1118.83 | 1124.83 | 1134.26 | 1112.51 | 1144.51 | 1194.77 | -6.32 | n.s | 1107.76 | 1147.76 | 1210.59 | -4.75 | n.s |
| 7 (default mode) | 8 (deep grey matter) | 1384.23 | 1390.23 | 1399.66 | 1372.14 | 1404.14 | 1454.40 | -12.09 | n.s | 1369.18 | 1409.18 | 1472.01 | -2.96 | n.s |
| *Within modules* | |  |  |  |  |  |  |  |  |  |  |  |  |  |
| 1 (visual) |  | 1107.03 | 1113.03 | 1122.46 | 1092.48 | 1124.48 | 1174.75 | -14.55 | n.s | 1090.84 | 1130.84 | 1193.68 | -1.64 | n.s |
| 2 (somatomotor) |  | 947.37 | 953.37 | 962.80 | 934.16 | 966.16 | 1016.42 | -13.21 | n.s | 929.87 | 969.87 | 1032.71 | -4.29 | n.s |
| 3 (dorsal attention) |  | 659.26 | 665.26 | 674.69 | 638.55 | 670.55 | 720.81 | -20.71 | n.s | 637.09 | 677.09 | 739.92 | -1.46 | n.s |
| 4 (ventral attention) |  | 626.07 | 632.07 | 641.49 | 613.15 | 645.15 | 695.42 | -12.92 | n.s | 610.95 | 650.95 | 713.78 | -2.20 | n.s |
| 5 (limbic) |  | 1246.29 | 1252.29 | 1261.72 | 1236.71 | 1268.71 | 1318.98 | -9.58 | n.s | 1230.37 | 1270.37 | 1333.20 | -6.34 | n.s |
| 6 (fronto-parietal) |  | 907.96 | 913.96 | 923.39 | 898.24 | 930.24 | 980.50 | -9.72 | n.s | 896.43 | 936.43 | 999.26 | -1.81 | n.s |
| 7 (default mode) |  | 1270.15 | 1276.15 | 1285.57 | 1255.95 | 1287.95 | 1338.22 | -14.20 | n.s | 1254.50 | 1294.50 | 1357.33 | -1.45 | n.s |
| 8 (deep grey matter) |  | 938.63 | 944.63 | 954.06 | 927.41 | 959.41 | 1009.68 | -11.22 | n.s | 919.82 | 959.82 | 1022.65 | -7.59 | n.s |

Model 0 = no fixed factors; Model 1 fixed factor = age; Model 2 fixed factor = age, group; ^a^ Significant effect for group (uncorrected) (HC > stage 1b).

Supplementary Table 2b. Linear mixed effects analysis for functional connectivity modular interactions (based on Yeo et al. 2011).

|  |  | Model 0 | AIC | BIC | Model 1 | AIC | BIC | *χ^2^* | Sig. (uncorrected) | Model 2 | AIC | BIC | *χ^2^* | Sig.  (uncorrected) |
| --- | --- | --- | --- | --- | --- | --- | --- | --- | --- | --- | --- | --- | --- | --- |
|  |  | -2LL |  |  | -2LL |  |  | *change* |  | -2LL |  |  | *change* |  |
| *Between modules* | |  |  |  |  |  |  |  |  |  |  |  |  |  |
| 1 (visual) | 2 (somatomotor) | 1591.36 | 1597.36 | 1606.78 | 1590.28 | 1600.28 | 1615.99 | -1.08 | n.s | 1588.29 | 1606.29 | 1634.57 | -1.99 | n.s |
| 1 (visual) | 3 (dorsal attention) | 1190.58 | 1196.58 | 1206.01 | 1185.63 | 1195.63 | 1211.34 | -4.95 | n.s | 1182.99 | 1200.99 | 1229.27 | -2.64 | n.s |
| 1 (visual) | 4 (ventral attention) | 1255.42 | 1261.42 | 1270.85 | 1252.28 | 1262.28 | 1277.98 | -3.14 | n.s | 1251.57 | 1269.57 | 1297.84 | -0.71 | n.s |
| 1 (visual) | 5 (limbic) | 1557.05 | 1563.05 | 1572.47 | 1556.61 | 1566.61 | 1582.31 | -0.44 | n.s | 1554.84 | 1572.84 | 1601.12 | -1.77 | n.s |
| 1 (visual) | 6 (fronto-parietal) | 1331.70 | 1337.70 | 1347.13 | 1325.56^a^ | 1335.56 | 1351.27 | -6.14 | 0.05 | 1322.40 | 1340.40 | 1368.67 | -3.16 | n.s |
| 1 (visual) | 7 (default mode) | 1552.25 | 1558.25 | 1567.67 | 1548.18 | 1558.18 | 1573.89 | -4.07 | n.s | 1543.07 | 1561.07 | 1589.34 | -5.11 | n.s |
| 1 (visual) | 8 (deep grey matter) | 1397.54 | 1403.54 | 1412.97 | 1394.54 | 1404.54 | 1420.24 | -3.00 | n.s | 1393.85 | 1411.85 | 1440.12 | -0.69 | n.s |
| 2 (somatomotor) | 3 (dorsal attention) | 1197.85 | 1203.85 | 1213.28 | 1196.25 | 1206.25 | 1221.96 | -1.60 | n.s | 1183.49^c^ | 1201.49 | 1229.77 | -12.76 | 0.05 |
| 2 (somatomotor) | 4 (ventral attention) | 1327.67 | 1333.67 | 1343.10 | 1323.66 | 1333.66 | 1349.37 | -4.01 | n.s | 1320.73 | 1338.73 | 1367.00 | -2.93 | n.s |
| 2 (somatomotor) | 5 (limbic) | 1553.94 | 1559.94 | 1569.36 | 1553.02 | 1563.02 | 1578.73 | -0.92 | n.s | 1549.10 | 1567.10 | 1595.38 | -3.92 | n.s |
| 2 (somatomotor) | 6 (fronto-parietal) | 1435.06 | 1441.06 | 1450.48 | 1432.66 | 1442.66 | 1458.36 | -2.40 | n.s | 1428.96 | 1446.96 | 1475.24 | -3.70 | n.s |
| 2 (somatomotor) | 7 (default mode) | 1432.60 | 1438.60 | 1448.02 | 1426.09^a^ | 1436.09 | 1451.80 | -6.51 | 0.05 | 1424.63 | 1442.63 | 1470.91 | -1.46 | n.s |
| 2 (somatomotor) | 8 (deep grey matter) | 1455.61 | 1461.61 | 1471.03 | 1453.64 | 1463.64 | 1479.35 | -1.97 | n.s | 1449.14 | 1467.14 | 1495.41 | -4.50 | n.s |
| 3 (dorsal attention) | 4 (ventral attention) | 914.20 | 920.20 | 929.62 | 913.00 | 923.00 | 938.71 | -1.20 | n.s | 909.99 | 927.99 | 956.27 | -3.01 | n.s |
| 3 (dorsal attention) | 5 (limbic) | 1161.66 | 1167.66 | 1177.09 | 1161.52 | 1171.52 | 1187.23 | -0.14 | n.s | 1159.69 | 1177.69 | 1205.97 | -1.83 | n.s |
| 3 (dorsal attention) | 6 (fronto-parietal) | 1089.90 | 1095.90 | 1105.33 | 1087.71 | 1097.71 | 1113.42 | -2.19 | n.s | 1084.83 | 1102.83 | 1131.10 | -2.88 | n.s |
| 3 (dorsal attention) | 7 (default mode) | 1125.44 | 1131.44 | 1140.86 | 1121.23 | 1131.23 | 1146.93 | -4.21 | n.s | 1117.37 | 1135.37 | 1163.64 | -3.86 | n.s |
| 3 (dorsal attention) | 8 (deep grey matter) | 995.40 | 1001.40 | 1010.82 | 991.80 | 1001.80 | 1017.51 | -3.60 | n.s | 988.07 | 1006.07 | 1034.34 | -3.73 | n.s |
| 4 (ventral attention) | 5 (limbic) | 1290.32 | 1296.32 | 1305.74 | 1288.80 | 1298.80 | 1314.51 | -1.52 | n.s | 1283.08 | 1301.08 | 1329.36 | -5.72 | n.s |
| 4 (ventral attention) | 6 (fronto-parietal) | 1221.04 | 1227.04 | 1236.46 | 1220.76 | 1230.76 | 1246.47 | -0.28 | n.s | 1218.59 | 1236.59 | 1264.86 | -2.17 | n.s |
| 4 (ventral attention) | 7 (default mode) | 1216.50 | 1222.50 | 1231.92 | 1207.98^b^ | 1217.98 | 1233.69 | -8.52 | 0.05 | 1206.82 | 1224.82 | 1253.10 | -1.16 | n.s |
| 4 (ventral attention) | 8 (deep grey matter) | 1212.04 | 1218.04 | 1227.47 | 1208.55 | 1218.55 | 1234.26 | -3.49 | n.s | 1203.44 | 1221.44 | 1249.72 | -5.11 | n.s |
| 5 (limbic) | 6 (fronto-parietal) | 1324.74 | 1330.74 | 1340.17 | 1315.43^a^ | 1325.43 | 1341.13 | -9.31 | 0.01 | 1313.73 | 1331.73 | 1360.01 | -1.70 | n.s |
| 5 (limbic) | 7 (default mode) | 1537.66 | 1543.66 | 1553.09 | 1534.12 | 1544.12 | 1559.83 | -3.54 | n.s | 1528.97 | 1546.97 | 1575.25 | -5.15 | n.s |
| 5 (limbic) | 8 (deep grey matter) | 1416.13 | 1422.13 | 1431.55 | 1413.36 | 1423.36 | 1439.07 | -2.77 | n.s | 1411.53 | 1429.53 | 1457.81 | -1.83 | n.s |
| 6 (fronto-parietal) | 7 (default mode) | 1372.42 | 1378.42 | 1387.85 | 1370.82 | 1380.82 | 1396.53 | -1.60 | n.s | 1369.33 | 1387.33 | 1415.61 | -1.49 | n.s |
| 6 (fronto-parietal) | 8 (deep grey matter) | 1277.30 | 1283.30 | 1292.73 | 1276.25 | 1286.25 | 1301.95 | -1.05 | n.s | 1272.12 | 1290.12 | 1318.39 | -4.13 | n.s |
| 7 (default mode) | 8 (deep grey matter) | 1379.50 | 1385.50 | 1394.93 | 1376.33 | 1386.33 | 1402.04 | -3.17 | n.s | 1371.76 | 1389.76 | 1418.03 | -4.57 | n.s |
| *Within modules* | |  |  |  |  |  |  |  |  |  |  |  |  |  |
| 1 (visual) |  | 1327.80 | 1333.80 | 1343.22 | 1325.52 | 1335.52 | 1351.22 | -2.28 | n.s | 1323.70 | 1341.70 | 1369.97 | -1.82 | n.s |
| 2 (somatomotor) |  | 1305.00 | 1311.00 | 1320.43 | 1301.36 | 1311.36 | 1327.07 | -3.64 | n.s | 1299.81 | 1317.81 | 1346.09 | -1.55 | n.s |
| 3 (dorsal attention) |  | 679.50 | 685.50 | 694.92 | 670.74^b^ | 680.74 | 696.45 | -8.76 | 0.05 | 658.23^b,c^ | 676.23 | 704.51 | -12.51 | 0.05 |
| 4 (ventral attention) |  | 821.63 | 827.63 | 837.05 | 820.00 | 830.00 | 845.71 | -1.63 | n.s | 816.16 | 834.16 | 862.44 | -3.84 | n.s |
| 5 (limbic) |  | 1359.43 | 1365.43 | 1374.85 | 1353.51 | 1363.51 | 1379.21 | -5.92 | n.s | 1343.50^b,c^ | 1361.50 | 1389.77 | -10.01 | 0.05 |
| 6 (fronto-parietal) |  | 1101.14 | 1107.14 | 1116.57 | 1098.04 | 1108.04 | 1123.74 | -3.10 | n.s | 1091.12 | 1109.12 | 1137.39 | -6.92 | n.s |
| 7 (default mode) |  | 1342.44 | 1348.44 | 1357.87 | 1341.96 | 1351.96 | 1367.67 | -0.48 | n.s | 1335.66 | 1353.66 | 1381.94 | -6.30 | n.s |
| 8 (deep grey matter) |  | 1004.57 | 1010.57 | 1020.00 | 995.73^b^ | 1005.73 | 1021.44 | -8.84 | 0.05 | 991.81 | 1009.81 | 1038.08 | -3.92 | n.s |

Model 0 = no fixed factors; Model 1 fixed factor = age, head movement; Model 2 fixed factor = age, group.  ^a^Significant effect for head movement; ^b^Significant effect for age; ^c^Significant effect for group (uncorrected; no significant pairwise group differences after Bonferronni corrections).
